# Decreased expression of SLC16A12 mRNA predicts poor prognosis of patients with clear cell renal cell carcinoma

**DOI:** 10.1097/MD.0000000000016624

**Published:** 2019-07-26

**Authors:** Jie Mei, Kehan Hu, Xiafeng Peng, Huiyu Wang, Chaoying Liu

**Affiliations:** a Department of Oncology, Wuxi People's Hospital Affiliated to Nanjing Medical University, Wuxi; b Department of Clinical Medicine, the First Clinical Medical College, Nanjing Medical University, Nanjing, China.

**Keywords:** ccRCC, immunohistochemistry, prognosis, SLC16A12, TCGA

## Abstract

Solute carrier family 16, member 12 (SLC16A12) is a highly -expressed protein in the kidney and has been reported to participate in the transport of creatine. However, the clinical values of SLC16A12 in clear cell renal cell carcinoma (ccRCC) have not been explored.

SLC16A12 RNA-seq data and clinical information were downloaded from the Cancer Genome Atlas (TCGA) database. We compared its expression in ccRCC and paracancerous tissues, then the result was further validated with our cohort. The impact on the clinical significance of SLC16A12 in ccRCC was also assessed.

Compared with paracancerous tissue, SLC16A12 was significantly downregulated in the tumor tissues both in mRNA and protein level. In TCGA cohort, SLC16A12 mRNA expression was associated with several clinicopathological parameters, including T stages (*P* < .001), M stages (*P* = .009), TNM stages (*P* < .001), and differentiated grades (*P* = .001). Kaplan–Meier analysis showed that the overall survival of patients with low expression of SLC16A12 mRNA was significantly worse than that of patients with high expression (*P* < .001). Furthermore, both univariate (*HR* = 0.371, *95%CI*: 0.269–0.513, *P* < .001) and multivariate (*HR* = 0.485, *95%CI*: 0.297–0.793, *P* = .004) Cox regression analyses suggested that low expression of SLC16A12 mRNA was an independent prognostic factor for patients with ccRCC.

Overall, we uncovered that decreased expression of SLC16A12 is a poor prognostic factor for patients with ccRCC. SLC16A12 might be a potential biomarker and therapeutic target in ccRCC.

## 1 Introduction

Renal cell carcinoma (RCC) is one of the most common malignant tumors in the urinary system. The American Cancer Society predicts that there will be more than 60,000 new cases and nearly 15,000 deaths of RCC in the United States in 2018.^[1]^ Being a heterogeneous tumor, RCC is classified into various subtypes based on the histological details along with its genetic abnormalities. Major subtypes include clear cell RCC (ccRCC), papillary RCC (pRCC), chromophobe RCC (chRCC), collecting duct RCC, and unclassified RCC.^[2,3]^ CcRCC is the most common subtype of RCC, accounting for approximately 70%–75% cases.^[2]^ RCC is commonly resistant to conventional chemotherapy and radiotherapy, so early surgical resection remains the preferred therapy.^[4]^ However, an amount number of patients with advanced cancer, whose prognosis is largely poor, are still lack of effective therapies to prevent the progression. Thus, it is of great significance to discover novel targets for the effective treatment for patients with RCC.

Solute carrier family 16, member 12 (SLC16A12) is located on 10q23.3, which encodes a transmembrane transport protein containing 486 amino acids. SLC16A12 is a highly expressed protein in the kidney and has been reported to participate in the transport of creatine.^[5,6]^ Previous researches have demonstrated that the dysregulated expression and mutation of SLC16A12 in gene level are associated with a syndrome combining juvenile cataract with microcornea and renal glucosuria.^[7,8]^ However, no study is currently available focusing on the prognostic value and clinicopathological correlation of SLC16A12 expression in ccRCC.

In the current study, we collected SLC16A12 RNA-seq data from the cancer genome atlas (TCGA) to compare its expression in ccRCC and paracancerous tissues. The protein expression level of SLC16A12 in ccRCC was validated using tissue microarray slides. The associations between SLC16A12 mRNA expression and prognostic parameters, as well as various clinicopathological characteristics were also analyzed. Overall, these data suggest that SLC16A12 functions as a potential tumor suppressor in ccRCC, which could be a therapeutic target in limiting the progression of ccRCC.

## 2 Methods and materials

### 2.1 Data mining in the online database

The level of SLC16A12 mRNA expression in human normal tissues were reviewed via using data generated by the Human Protein Atlas (HPA) (http://www.proteinatlas.org/).^[9,10]^ The level 3 data in TCGA-KIRC and clinical information were downloaded from the UCSC Xena browser (https://xenabrowser.net). This database included RNA-seq data of 533 ccRCC tissues and 72 paracancerous tissues.

### 2.2 Data processing

A total of 528 cases containing both SLC16A12 mRNA expression data and clinical information was reserved to further analysis. According to the expression of SLC16A12 mRNA, the cases in the database were ranked from high expression to low expression. The top 50% patients were divided into the high expression group and the bottom 50% belonged to the low expression group.

### 2.3 Tissue samples

Tissue microarray slides (HKidCRCC150CS01) were purchased from Outdo Biotech (Shanghai, China, http://www.superchip.com.cn/). The slides contained 75 ccRCC tissues and paired normal renal specimens. All ccRCC tissues were histopathologically confirmed by a pathologist who selected areas of higher tumor cell density for hematoxylin-eosin (HE) staining. Ethical approval for study of tissue microarray slides was granted by the Clinical Research Ethics Committee, Outdo Biotech (Shanghai, China).

### 2.4 Immunohistochemistry (IHC)

IHC staining was performed directly on the tissue slides. The primary antibodies were as following: anti-SLC16A12 (1:200 dilution, Cat. 20553-1-AP, ProteinTech, Wuhan, China). Antibody staining was visualized with DAB and hematoxylin counterstain (ZSGB-Bio). The percentage of positively stained cells was scored on a scale of 0 to 4 as follows: 0 (<1%), 1 (1%–25%), 2 (25%–50%), 3 (50%–75%) and 4 (>75%). The staining intensity was scored from 0 to 3 as follows: 0 (negative), 1 (weak), 2 (moderate), and 3 (strong). Quantitative analysis of the staining was performed based on the percentage of positive cells and staining density by 2 independent pathologists using 12 standard points. Immunostained sections were scanned using a microscope (Olympus Corporation, Tokyo, Japan).

### 2.5 Statistical analysis

Statistical analysis was performed by using SPSS 25.0 (Chicago, IL). Most of the data were analyzed by Student *t* test or one-way ANOVA followed by Dunnett's multiple posthoc tests. The significance of correlations between the clinicopathological characteristics and SLC16A12 mRNA expression was performed using *χ*^2^ tests. Log-rank test was performed to assess the difference between the survival curves. Prognostic values were analyzed by univariate and multivariate Cox regression models. Bar charts show means ± SDs if not noted. For all analyses, a 2-sided *P* value of less than .05 was considered statistically significant.

## 3 Results

### 3.1 SLC16A12 mRNA was highly expressed in normal kidney tissues

**Figure 1:**
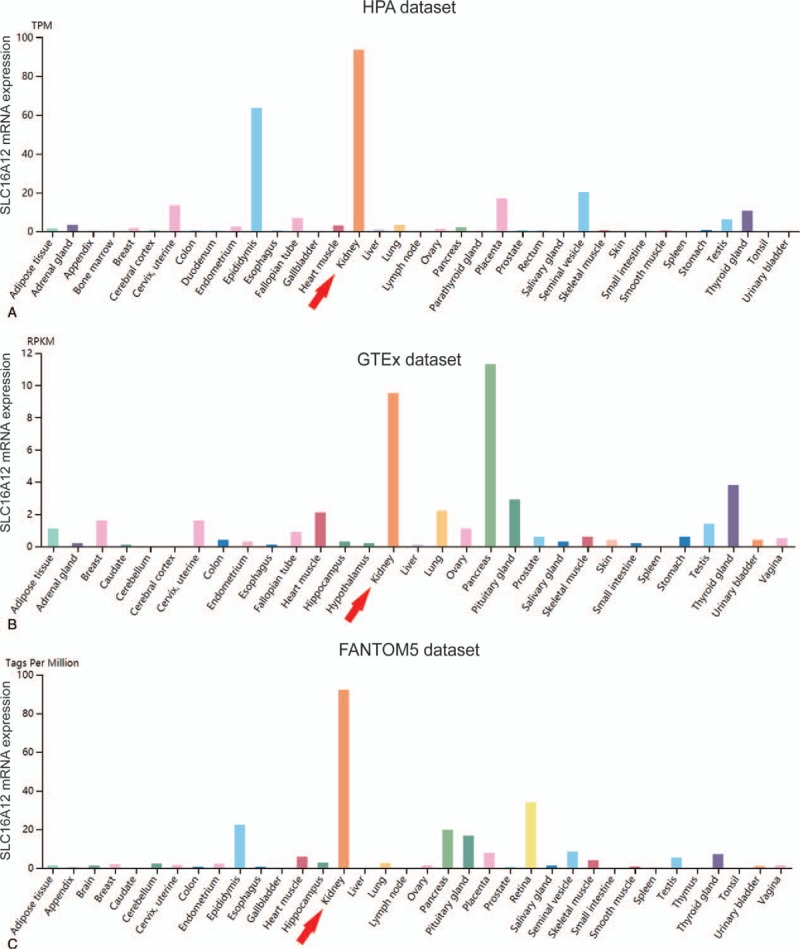
SLC16A12 mRNA expression profiles in normal human tissues. The mRNA expression profiles of SLC16A12 in normal human tissues in (A) HPA dataset, (B) GTEx dataset, and (C) FANTOM5 dataset. SLC16A12 mRNA was significantly highly expressed in normal kidney tissues compared with most other tissues. Data summary images could be obtained from Human Protein Atlas via visiting the website: https://www.proteinatlas.org/ENSG00000152779-SLC16A12/tissue.

Several studies have implied that SLC16A12 is a specifically kidney-expressed protein.^[6,11]^ At first, we examined whether SLC16A12 was enriched in normal kidney tissues. After reviewing the RNA-seq data in the HPA, we characterized SLC16A12 mRNA expression profiles in various normal human tissues. As shown in Figure 1, the mRNA expression of SLC16A12 was significantly highly expressed in normal kidney based on three independent datasets, including HPA dataset (Fig. 1A), GTEx dataset (Fig. 1B), and FANTOM5 dataset (Fig. 1C).

### 3.2 SLC16A12 expression was decreased at both mRNA and protein level in ccRCC

**Figure 2:**
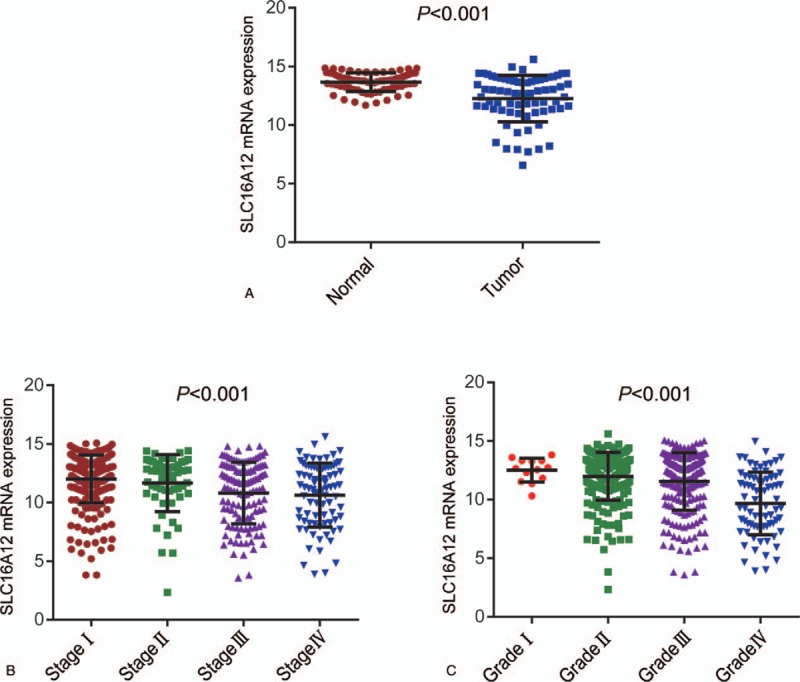
SLC16A12 mRNA expression in ccRCC tissues and paired normal kidney tissues based on TCGA database. (A) Expression of SLC16A12 mRNA in ccRCC tissues and paired normal kidney tissues (n = 72) according to the TCGA database. Compared with the paired normal kidney tissues, ccRCC tissues expressed notably low SLC16A12 mRNA (*P* < .001). (B, C) Expression of SLC16A12 mRNA among various clinicopathological groups. SLC16A12 mRNA expressions were compared among the various TNM stages and pathological differentiated grades. The one-way ANOVA was used to analyze the differences between SLC16A12 mRNA expression and various TNM stages and pathological differentiated grades in ccRCC patients.

**Figure 3:**
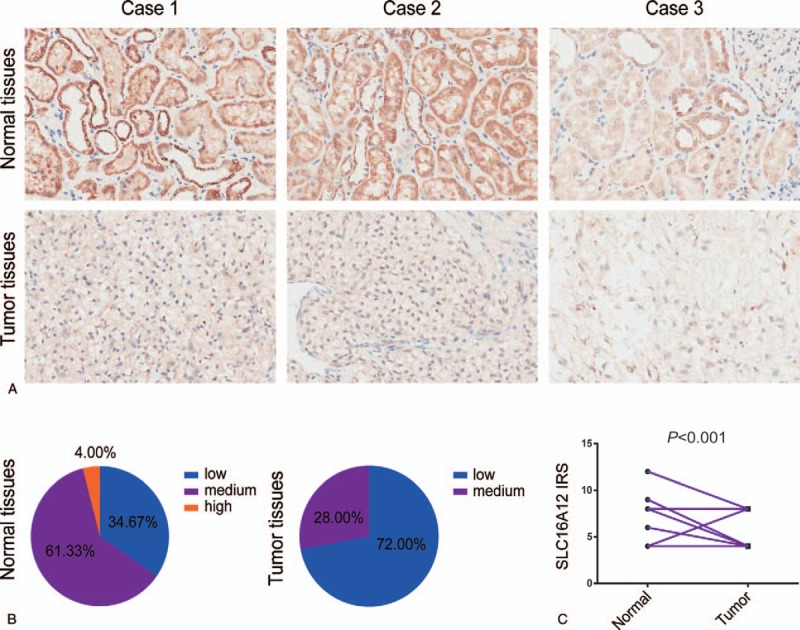
SLC16A12 expression in ccRCC tissues and normal kidney tissues in the validated cohort. (A) Staining of SLC16A12 was evaluated by an immunoreactivity score (IRS) by multiplying the intensity score with the score of percentage of positive cells. Representative microphotographs of 3 cases including ccRCC tissues and paired normal tissues. Brown, SLC16A12. Blue, hematoxylin. Original magnification ×200. (B) SLC16A12 protein expression intensity proportion of ccRCC tissues and paired normal tissues. Low expression: IRS ≤ 4; Medium expression: 4 < IRS ≤ 8; High expression: IRS > 8. (C) The expression of SLC16A12 protein in ccRCC tissues and paired normal tissues. A significant decrease of SLC16A12 expression was observed in ccRCC tissues compared with paired normal tissues.

In view of the fact that SLC16A12 was highly expressed in kidney, we next examine the expression of SLC16A12 mRNA in ccRCC tissues. The expression of SLC16A12 mRNA in normal and tumor tissues were compared in TCGA-KIRC database, compared to the normal kidney tissues, SLC16A12 mRNA expression was significantly downregulated in tumor tissues (*P* < .001, Fig. 2A). Furthermore, we found that expression of SLC16A12 mRNA was notably decreased in ccRCC tissues associating with TNM stages (*P* < .001, Fig. 2B) and differentiated grades (*P* < .001, Fig. 2C) negatively. Moreover, the protein abundance of SLC16A12 in normal and tumor tissues was also quantified by IHC for validation. As shown in Figure 3A, the immunoreactivity of SLC16A12 was localized mostly in the cytoplasm. After the analysis of 75 ccRCC cases, we demonstrated that the sections lowly and moderately expressed SLC16A12 accounted for a majority of tumor tissues (Fig. 3B). Besides, the IRS of SLC16A12 in the tumor tissues was notably lower than paired normal tissues (*P* < .001, Fig. 3C). All these results above suggest SLC16A12 was downregulated in ccRCC tissues.

### 3.3 SLC16A12 mRNA expression was correlated with clinicopathological characteristics in ccRCC

Next, the association between clinicopathological characteristics and SLC16A12 mRNA expression was evaluated in TCGA-KIRC cohort. As shown in Table 1, the expression of SLC16A12 mRNA was not associated with gender (*P* = .144) and age (*P* = .296), However, SLC16A12 mRNA was significantly decreased in the patients with advanced T stages (*P* < .001), distant metastasis (*P* = .009), TNM stages (*P* < .001), and differentiated grades (*P* = .001). In other words, the expression of SLC16A12 mRNA was negatively associated with malignant tendency of the tumor (Table 2).

**Table 1:**
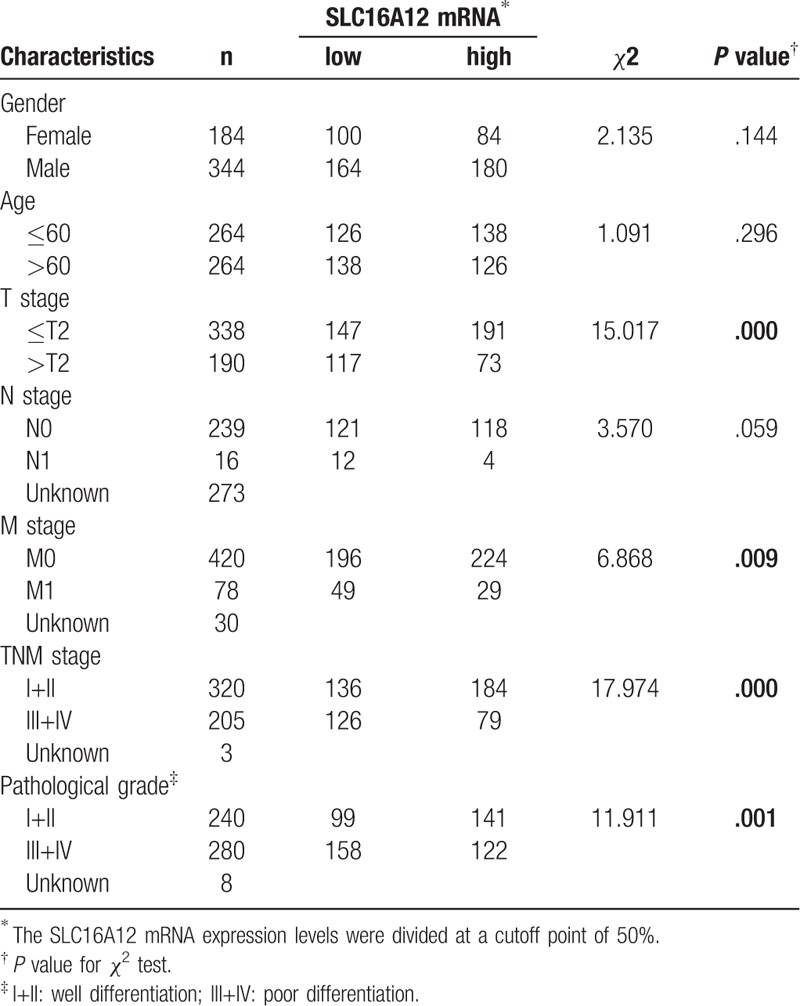
Association between SLC16A12 mRNA expression and patients’ characteristics in ccRCC.

**Table 2:**
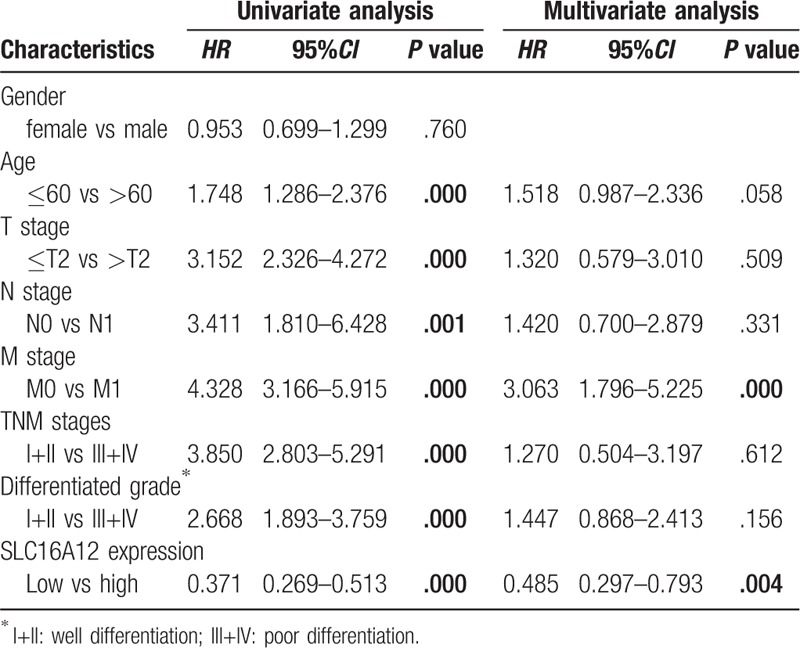
Univariate and multivariate analysis of survival in patients with ccRCC.

### 3.4 Downregulated SLC16A12 mRNA expression was associated with poor prognosis in ccRCC

**Figure 4:**
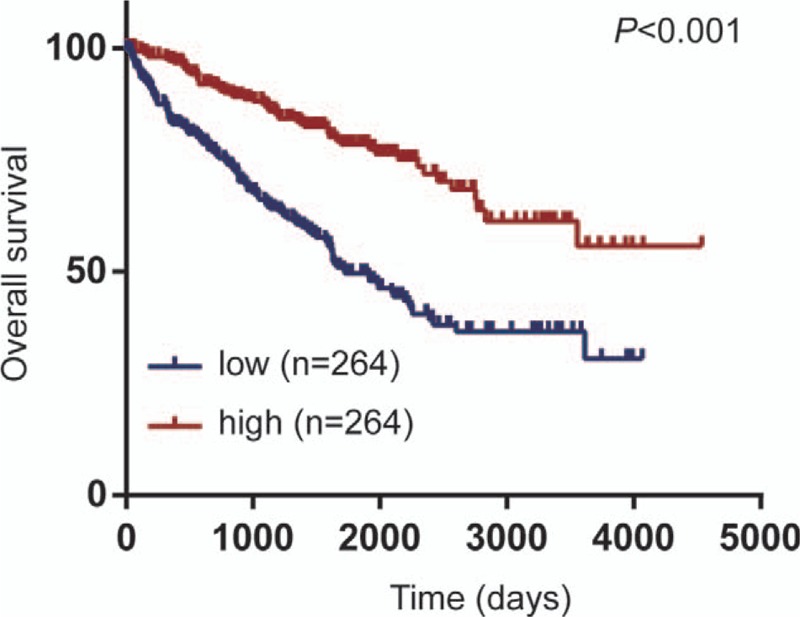
Prognostic value of SLC16A12 mRNA expression in ccRCC patients. Downregulated SLC16A12 mRNA expression was associated with poor prognosis in ccRCC patients (*P* < .001). The SLC16A12 mRNA expression levels were divided at a cutoff point of 50%.

**Figure 5:**
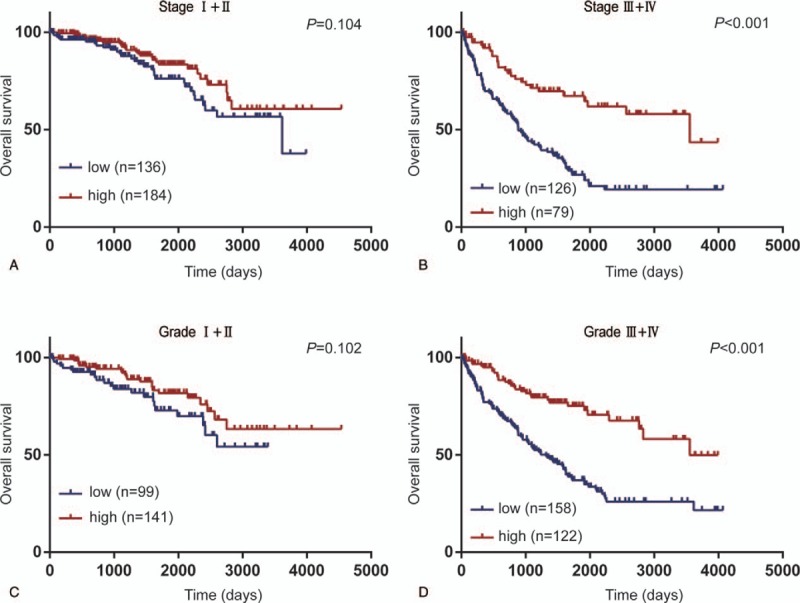
Subgroup analysis of the prognostic value of SLC16A12 mRNA in patients ccRCC. (A, B) The decreased expression of SLC16A12 mRNA was not associated with OS (*P* = .104) in patients with TNM stage I and II, whereas in patients with TNM stage III and IV, the OS was significantly lower in patients with low SLC16A12 mRNA expression compared with high expression group (*P* < .001). (C, D) Subgroup analysis by pathological grade showed that the expression of SLC16A12 mRNA was not associated with OS in patients with grade I and grade II (*P* = .102), but in the poorly differentiated group, including grade III and IV group, the OS of patients with SLC16A12 mRNA low expression group was notably lower than that of high expression group (*P* < .001). The SLC16A12 mRNA expression levels were divided at a cutoff point of 50%. OS = overall survival.

The Kaplan–Meier method was employed to investigate the correlation between SLC16A12 mRNA expression and overall survival (OS) in 528 patients with ccRCC. The results showed that the OS of patients with low SLC16A12 mRNA expression was notably worse than high expression group (*P* < .001, Fig. 4). Further subgroup analysis of the patients’ TNM stages suggested that the expression of SLC16A12 mRNA was not correlated with OS in patients with stage I and II (*P* = .104, Fig. 5A), whereas in patients with stage III and IV, the OS of patients with low SLC16A12 mRNA expression was notably worse than high expression group (*P* < .001, Fig. 5B). In addition, subgroup analysis by differentiated grades showed that the expression of SLC16A12 mRNA was not associated with OS in patients with grade I and grade II (*P* = .102, Figure 5C), but in the poorly differentiated group, including grade III and IV, the OS of the SLC16A12 mRNA low expression group was significantly worse than high expression group (*P* < .001, Fig. 5D).

### 3.5 Univariate and multivariate analyses of prognostic factors for ccRCC

To further assess the prognostic factors of patients with ccRCC, the Cox regression model was used for univariate and multivariate survival analyses. Univariate analysis showed that age (*HR* = 1.748, *95%CI*: 1.286–2.376, *P* < .001), T stages (*HR* = 3.152, *95%CI*: 2.326–4.272, *P* < .001), N stages (*HR* = 3.411, *95%CI*: 1.810–6.428, *P* = .001), M stages (*HR* = 4.328, *95%CI*: 3.166–5.915, *P* < .001), TNM stages (*HR* = 3.850, *95%CI*: 2.803–5.291, *P* < .001), differentiated grades (*HR* = 2.668, *95%CI*: 1.893–3.759, *P* < .001) and SLC16A12 mRNA expression (*HR* = 0.371, *95%CI*: 0.269–0.513, *P* < .001) were associated with OS. In multivariate analysis, we found that M stages (*HR* = 3.063, *95%CI*: 1.796–5.225, *P* < .001) and SLC16A12 mRNA expression (*HR* = 0.485, *95%CI*: 0.297–0.793, *P* = .004) were independent prognostic factors for ccRCC.

## 4 Discussion

Increasing evidence shows that members of the SLC16A family participate in carcinogenesis and progression of various cancers.^[12–15]^ The SLC16A family consists of 14 members and is mainly involved in the regulation of transmembrane transport of monocarboxylic acids, such as lactic acid, pyruvic acid, butyric acid, etc, which is significant for glycolysis metabolism of both normal cells and tumor cells, especially lactic acid metabolism. SLC16A1, SLC16A2, SLC16A3, and SLC16A4 are H^+^-coupled monocarboxylic acid transporters, SLC16A8 and SLC16A10 transport thyroid hormones and aromatic amino acids.^[6]^ However, the specific substrate and function of other SLC16As have not been fully determined. Several researchers have revealed that SLC16As are dysregulated in a variety of tumors, and the dysregulated expression is generally considered to be closely related to lactic acid metabolism of tumor cells.^[16,17]^

SLC16A12, a member of the SLC16A family, whose specific substrate and function are largely unknown. A few studies have preliminarily evaluated the role of SLC16A12 in the progression of tumors. Chung et al revealed that DNA hypermethylation of SLC16A12 in CpG island was observed in colon cancer, breast cancer, and prostate cancer tissues compared paracancerous tissues.^[18]^ Tahara et al suggested that DNA methylation accumulation of SLC16A12 is associated with gastric carcinogenesis after *Helicobacter pylori* eradication.^[19]^ Hypermethylation of CpG island in the promoter region has been identified as an important cause for downregulation of gene expression.^[20,21]^ So, the phenomenon that hypermethylation of SLC16A12 suggests that SLC16A12 might be a critical tumor suppressor. However, no exact researches on the expression and mechanism of SLC16A12 in tumors were available up to now.

In this study, we determinated the expression of SLC16A12 mRNA based on TCGA cohort, significantly decreased expression of SLC16A12 mRNA was found in ccRCC tissues compared with paired normal tissues. Besides, downregulated SLC16A12 mRNA expression was correlated with T stages (*P* < .001), M stages (*P* = .009), TNM stages (*P* < .001), and differentiated grades (*P* = .001). Our results also suggest that the crucial role in the prognosis of ccRCC patients. Kaplan–Meier analysis showed that the OS of patients with low expression of SLC16A12 mRNA was significantly worse than that of patients with high expression (*P* < .001). Interestingly, subgroup analysis revealed that the prognostic value of SLC16A12 mRNA expression was more notable in patients with higher TNM stages and poorer differentiated grades. In addition, both univariate and multivariate Cox regression analyses suggested that low expression of SLC16A12 mRNA was an independent prognostic factor for the prognosis of patients with ccRCC.

To the best of our knowledge, this is the first research to evaluate the association between SLC16A12 expression and clinicopathological parameters for patients with ccRCC. Given to all the findings, we suggest that SLC16A12 may play an important role of tumor suppressor in the carcinogenesis of ccRCC, and that this may be a potential target to limit the progression of ccRCC. Besides, the determination of the SLC16A12 expression may serve as a biomarker to predict the prognosis of patients with ccRCC.

## 5 Conclusion

To conclude, we found that a substantial decrease of SLC16A12 expression in ccRCC tissues based on TCGA cohort and validation of tissue microarray. Our findings strongly suggest that SLC16A12 mRNA has significant value in predicting the outcome of patients and may be a novel biomarker in ccRCC. Furthermore, our promising results encourage further studies of the mechanisms by which SLC16A12 suppresses carcinogenesis and progression of ccRCC.

## Author contributions

**Conceptualization:** Jie Mei.

**Data curation:** Jie Mei.

**Formal analysis:** Jie Mei, Xiafeng Peng.

**Funding acquisition:** Huiyu Wang, Chaoying Liu.

**Investigation:** Jie Mei, Kehan Hu.

**Methodology:** Jie Mei, Kehan Hu, Xiafeng Peng, Huiyu Wang.

**Project administration:** Huiyu Wang, Chaoying Liu.

**Validation:** Kehan Hu, Chaoying Liu.

**Writing – original draft:** Jie Mei.

**Writing – review & editing:** Jie Mei, Huiyu Wang, Chaoying Liu.
